# A road-map for addressing antimicrobial resistance in low- and middle-income countries: lessons learnt from the public private participation and co-designed antimicrobial stewardship programme in the State of Kerala, India

**DOI:** 10.1186/s13756-020-00873-9

**Published:** 2021-02-11

**Authors:** Sanjeev Singh, Esmita Charani, Sarada Devi, Anuj Sharma, Fabia Edathadathil, Anil Kumar, Anup Warrier, P. S. Shareek, A. V. Jaykrishnan, K. Ellangovan

**Affiliations:** 1 Amrita Institute of Medical Sciences, Amrita Vishwavidyapeetham, Kochi, India; 2 NIHR Health Protection Research Unit in Healthcare Associated Infections and Antimicrobial Resistance, Imperial College, London, UK; 3 Trivandrum Medical College, Trivandrum, India; 4 Technical Focal Point, Antimicrobial Resistance, Laboratory and Infection Prevention and Control, WHO Country Office, Delhi, India; 5 Aster Medcity Hospital, Kochi, India; 6 Sree Uthradom Thirunal Hospital, Trivandrum, India; 7 Indian Medical Association, Kochi, India; 8 Ministry of Health and Family Welfare, Government of Kerala, Trivandrum, India

**Keywords:** Antimicrobial stewardship, Public private partnership, Antimicrobial resistance, Kerala model, KARSAP

## Abstract

**Background:**

The global concern over antimicrobial resistance (AMR) is gathering pace. Low- and middle-income countries (LMICs) are at the epicentre of this growing public health threat and governmental and healthcare organizations are at different stages of implementing action plans to tackle AMR. The South Indian state of Kerala was one of the first in India to implement strategies and prioritize activities to address this public health threat.

**Strategies:**

Through a committed and collaborative effort from all healthcare related disciplines and its professional societies from both public and private sector, the Kerala Public Private Partnership (PPP) has been able to deliver a state-wide strategy to tackle AMR A multilevel strategic leadership model and a multilevel implementation approach that included developing state-wide antibiotic clinical guidelines, a revision of post-graduate and undergraduate medical curriculum, and a training program covering all general practitioners within the state the PPP proved to be a successful model for ensuring state-wide implementation of an AMR action plan. Collaborative work of multi-professional groups ensured co-design and development of disease based clinical treatment guidelines and state-wide infection prevention policy. Knowledge exchange though international and national platforms in the form of workshops for sharing of best practices is critical to success. Capacity building at both public and private institutions included addressing practical and local solutions to the barriers e.g. good antibiotic prescription practices from primary to tertiary care facility and infection prevention at all levels.

**Conclusion:**

Through 7 years of stakeholder engagement, lobbying with government, and driving change through co-development and implementation, the PPP successfully delivered an antimicrobial stewardship plan across the state. The roadmap for the implementation of the Kerala PPP strategic AMR plan can provide learning for other states and countries aiming to implement action plans for AMR.

## Background

The growing threat of antimicrobial resistance (AMR) is a major public health challenge, leading to complications in infectious disease management and contributing to high patient morbidity, mortality and cost of care [1]. Deaths attributable to AMR are currently estimated to be 700,000 per year globally with 99,000 hospital acquired infection (HAI) related deaths occurring in the USA alone [2, 3]. The economic burden in terms of health services costs and productivity loss has been reported to be $55 billion in USA annually and the global economic loss has been projected to be $100 trillion by 2050, if left unchecked [4]. The O’Neill report on AMR has estimated that up to 90% of the 10 million projected deaths associated with AMR will occur in low- and middle-income countries (LMICs) [4].

Amongst the LMICs, India bears a significant burden of AMR-related infection (Table 1) [5]. The Indian clinical laboratory susceptibility data report alarming rates of AMR among major pathogens including *Enterococcus faecium, Staphylococcus aureus, Klebsiella pneumoniae, Acinetobacter baumannii, Pseudomonas aeruginosa*, and *Enterobacter* spp., as well as a rise in resistance to commonly used antibiotics for treating *Salmonella*, *Shigella, Vibrio cholerae, Neisseria gonorrhoeae and Mycobacterium tuberculosis* [6, 7]. AMR rates in India are accentuated by emergence of *AmpC, New Delhi metallo-beta-lactamases, Vancomycin-resistant Enterococci and* Extensively Drug Resistant (XDR) *Mycobacterium tuberculosis* [8]. Mortality associated with colistin and carbapenem resistant Klebsiella is high in India [9] and over 30% of neonatal sepsis deaths are attributable to AMR [10]. A recent study from North Indian reported a survival rate of 56.5% among neonates with MDR sepsis [11]. Pneumonia alone results in 410,000 deaths annually among children aged less than 5 years and 70% of pathogens causing pneumonia and sepsis show broad spectrum AMR [12].

**Table 1:** Antimicrobial resistance profiles of major pathogens across countries

Reported aggregate resistance rates (from isolates from blood and cerebrospinal fluids from inpatients of all ages) among prevalent pathogens, by antibiotic class [5]^a^	India (%)	USA (%)	South Africa (%)	China (%)
*Gram negative*				
Carbapenem resistant *Klebsiella pneumoniae*	59	3	7	36
Fluoroquinolone resistant *Escherichia coli*	84	31	28	56
Carbapenem resistant *Acinetobacter baumanni*	77	30	73	82
*Gram positive*				
Oxacillin resistance of *Staphylococcus aureus*	39	45	27	38
Aminoglycosides resistant *Enterococcus faecalis*	60	34	50	31

^a^Antimicrobial susceptibility data procured from Centre for Disease Dynamics Economics and Policy (CDDEP). The resistance data rely on different testing methodologies across the different countries

In addition to this overwhelming AMR burden, the country leads in antimicrobial consumption with high use of broad spectrum agents, including penicillins which have a Drug Resistance Index (DRI) of 71.6 [13]. DRI is a single, standardized composite measure that reflects weighted aggregate of antibiotic consumption and drug resistance across different species [13].

### The national response to AMR in India

In India, inappropriate antibiotic use and over the counter sale of drugs are one of the key drivers of AMR, in addition to lack of universal access to water, sanitation and hygiene (WASH) [14]. Several policy interventions to address AMR were initiated at the national level from 2010 onwards, including the formation of a National Task Force on AMR Containment, development of a national policy for containment of AMR and integration of AMR containment as a national programme in the 12^th^ five-year plan in 2011. The Chennai declaration of 2012 had a positive impact in shaping national AMR strategies by engaging medical associations and societies for AMR containment. The Indian Council of Medical Research established AMR surveillance centres throughout the country [15, 16]. In India legislation pertaining to prescription only medicines, including antimicrobials is under Schedule H category. In 2013, a new Schedule, H1, was created under the schedule H category medicines to regulate the newer class of antimicrobials. Schedule H1 imposed conditions for the sale of antibiotics with an aim to control their inappropriate and excessive use as part of National policy for containment of antimicrobial resistance in India. Since then, a decline in antimicrobial sales belonging to Schedule H1 category has been reported, although a corresponding increase in the sales of drugs in non-schedule H1 category medicines has been observed as a ‘substitution effect’ [17]. The launch of the ‘Red Line Campaign’ in 2016 introduced public awareness on antimicrobial misuse [8]. Taking a leaf from hard-hitting messages used on tobacco packaging, the campaign was effective in raising awareness on the dangers of misusing antibiotics [16, 17]. A National Action Plan on AMR (NAP-AMR) was formulated by the Indian Ministry of Health and Family Welfare in 2016 that was endorsed in April 2017, detailing a five-year plan of six strategic priorities and interventional policies for combating AMR in the country [19]. As part of the national drive, the One Health approach has been incorporated into the NAP-AMR objectives, encompassing human health, agriculture, animal husbandry, fisheries, food processing units and the environment.

Antimicrobial Stewardship (AMS), is recognised as a key strategic intervention to reduce AMR in India’s NAP-AMR with demonstrated success in curbing AMR rates and reducing economic costs [15, 18]. The existence of AMS teams in healthcare organizations, however, is reported to be at a rudimentary stage across India except in selected public and private centres where AMS has demonstrated positive outcomes [19, 20]. Efforts to strengthen the AMR response and AMS among healthcare centres across the country the Indian Council of Medical Research (ICMR) has initiated a series of regional workshops to develop local capacity. Despite existing efforts, much work remains in India to develop a national strategy for tackling AMR and implementing AMS.

### The healthcare system in Kerala

The healthcare system in Kerala has been a culmination of resource redistribution policies and several decades long social investments in primary healthcare and universal education along with empowerment of women [21–23]. With a high (0.779) Human Development Index compared to rest of the country (0.647 for India) Kerala [24, 25] offers universal coverage for public healthcare and has achieved low infant mortality rates at 10/1000 live births (the national rate is 34/1000 live births) and higher life expectancy at birth (74 years for Kerala versus 69.16 for India) under a moderate economic landscape [26–28]. The three tier medical system of the state comprising of modern and alternate medical practices amounts to over 2700 institutions in the government sector alone, accounting for a bed to population ratio of 8.78 and average Doctor to hospital Bed Ratio of 6.95, excluding the substantial private healthcare sector [30]. The health system is a combination of public healthcare, private insurance and out of pocket expenditure models [31].

The heightened awareness and health seeking behaviours set in motion by high education levels provides a conducive environment for public healthcare endeavours at all levels of care. Various global reports on AMR policies have stressed the lack of collaborative efforts and emphasize the importance for a co-ordinated action in a political context to drive strategies to combat AMR [32–34]. Identifying health as infrastructure and a state policy for public private partnerships (PPP), several thriving PPPs have been implemented in Kerala including a Revised National Tuberculosis Program (RNTCP) nodal networks, and an initiative by private hospitals actively participating in community medical care [35, 36]. Public health emergencies such the Nipah virus outbreak in 2018, the ongoing novel coronavirus disease (COVID-19) pandemic and seasonally recurrent neglected tropical diseases, have tested states’ outbreak control and containment strategy for infectious disease epidemics [37–39]. Critical lessons in public health have been learnt from the handling of other local epidemics, including the Nipah virus outbreak. A grassroots approach to public health was exercised with success in that outbreak, through consistent public health messages delivered at community level and steered at state level, with strategic co-ordination through central government and collaboration with local government. The public health system consistently is able to efficiently reach grassroot level through a network of empowered women known as Accredited Social Health Activist (ASHA) who are trained community care workers [40]. Additionally community leadership was employed to disseminate the public health strategies of the central and local health authorities, leading to formal and informal routes of influence to deliver the public health strategy. Critically the public health response was initiated from the onset, after the first index cases were reported.

### The road map for a PPP model to tackle AMR

**Fig. 1:**
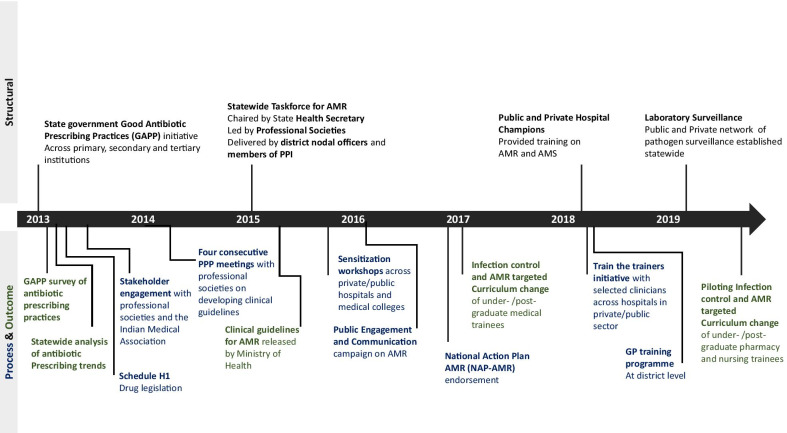
A road map of the state-wide antimicrobial stewardship plan to tackle AMR in Kerala

The increasing burden of AMR and antibiotic prescribing was the driving force for developing a state-wide PPP for AMR. Learning form lessons of other infectious disease public health interventions, and through its PPP, the State of Kerala was the first in the country to formulate the Kerala Antimicrobial Resistance Strategic Action Plan (KARSAP) for combating AMR in 2018 incorporating One Health. Like the Nipah public health campaign the KARSAP was overseen by the State Government, supported by the professional bodies, led by the PPP, and delivered through grassroots initiative involving champions, nodal officers and PPP representatives (Fig. 1). The partnership and its objectives were achieved through seven years of stakeholder engagement, including government, and driving change through co-development and implementation. Critically the KARSAP adopted structural change through formal channels as well as implementing process and outcome measures to deliver the AMR strategy.

The key features of the PPP initiative were to ensure the establishment of structural, as well as process and outcome initiatives to ensure the top down accountability as well as bottom up sustainability. The State Government advised by the PPP, initiated and scaled up Good Antibiotic Prescription Practices (GAPP) across all primary, secondary and tertiary care institutions within the State [41, 42]. The GAPP initiatives spanned all private medical colleges and private hospitals as well as the government funded ones.

The impetus for change at state level was accelerated following a government led survey among general practitioners to understand the baseline antibiotic prescription practices. The survey identified key gaps including lack of knowledge amongst the respondents about the mechanism of action of antibiotics, and the pressure by patients to be prescribed antibiotics. These results led to the formation of a State Wide Task Force along with a formation of a Core Committee at the State level. A State Core Committee was convened with representation from key stakeholders to identify the steps in addressing AMR. The Committee interacted with all the major stakeholders and supervised action items decided at State Task Force meetings. In line with the scale of the program, it was decided to engage with all State chapter of professional societies. The senior office bearers of 14 professional bodies were contacted and they all agreed to participate under the leadership of IMA State chapter (a list of all participating professional bodies is provided in Table 2).

**Table 2:** Professional Bodies who participated towards formulating Antibiotic Clinical Guidelines and IPC Guidelines

No		Professional societies
1	DHS, Kerala	District Health Services
2	DME, Kerala	Director of Medical Education
3	NHM, Kerala	National Health Mission, Kerala
4	KMSCL	Kerala Medical Services Corporation Limited
5	Govt MC	All Government Medical Colleges
6	ESI	Employee State Insurance Hospitals
7	Private MC	All Private Medical Colleges
8	IMA, Kerala	Indian Medical Association, Kerala
9	AOI	Association of Otorhinolaryngologists, Kerala Chapter
10	API	Association of Physicians of India, Kerala Chapter
11	ASI	Association of Surgeons of India, Kerala Chapter
12	KSOS	Kerala State Ophthalmic Surgeons
13	IDA	Indian Dental Association, Kerala Chapter
14	KFOG	Kerala Federation of OBGYN
15	ACM	Association of College of Microbiologists, Kerala Chapter
16	IPHA	Indian Public Health Association, Kerala Chapter
17	IOA	Indian Orthopaedics Association, Kerala Chapter
18	ICS	Indian Chest Society, Kerala Chapter
19	KGMOA	Kerala Govt Medical Officers Association
20	KGMCTA	Kerala Govt Medical College Teachers Association
21	QPMPA	Qualified Medical private Practitioners Association

In collaboration with state physicians’ association, at the hospital level, across tertiary private and government hospitals and the public health department, ‘Training the Trainers’ (TT) sessions were rolled out for all physicians to sensitize them about the state antimicrobial policy and rational prescribing of antibiotics. As part of the TT sessions, a structured multiple choice questionnaire comprising of 30 questions addressing appropriate antibiotic use in general and accurate diagnosis in specific infectious diseases case scenarios was disseminated before and after the TT session among the physicians. AMR surveillance was established in PPP mode for 6 pathogens of AMR concern and for major foci of infections (Fig. 1).

The PPP identified state level action plan including specific strategies to combat AMR which were adopted across the healthcare organisations (Box 1). The action plan was co-developed through consecutive meetings of the Committee with core stakeholders, including the professional bodies. The state-wide Antibiotic Clinical Guidelines clinical were developed through the Committee and the private institutions who had capacity conducted regular training among faculty and staff in their respective organisations as part of the implementation of the guidelines. Each private institution was also encouraged to mentor five private institutions closer to their organisations in IPC and AMS. Private organisations participated in National Accreditation Board for Hospitals (NABH). The Kerala Antimicrobial Resistance Strategic Action Plan (KARSAP) was an important milestone towards combating AMR in Kerala, which was developed in association with the WHO India Country Office [43]. In line with its integration of One Health approach in AMR containment, the broad aims of KARSAP include awareness generation, AMR surveillance through laboratory data, infection prevention and control and facilitating research projects for AMR by involving all stakeholders from animal husbandry, agriculture, food, environment, research and civil society. Participation from private sector and professional bodies has been encouraging towards drafting the policy and training the trainers.

**Box 1:** State of Kerala Action Plan for Combating AMR Task Force [46]

1. The Antibiotic prescription guidelines & infection control policy to be available to the public through all official websites of National Health mission, Directorates of Health & Medical Education
2. Formation of a State and district level Task Force with designated State & District Nodal Officers with members from public/ private hospitals and professional bodies. The Task force to ensure the adoption and implementation of the policy at all levels
3. Organizing sensitization workshops across all public/private medical colleges, district/ taluk hospitals and selected private hospitals for sensitizing medical practitioners, pharmacists and other stake holders regarding the short term and long term objectives of antibiotic stewardship
4. Widespread information, education & communication (IEC) among public regarding the bad effects of self-medication & irrational antibiotic consumption
5. Strong Regulatory efforts through Pharmacy Council and Drug control Department to prevent the illegal dispensing of antibiotics without Standard Prescriptions
6. Workshops to be organised with deliberations by experts for all house surgeons who are passing out of Medical Colleges every year regarding the rational antibiotic usage & emerging AMR as a strategy of “catch them young for the better future”
7. Monitoring mechanism through quarterly review, wherein, every stakeholder has to report on the extent of the adoption and implementation of State antibiogram in their respective discipline
8. Review suggestions to be incorporated as the discussion points for the annual CME on state antibiogram and shall be published as a subsequent version
9. Regular CMEs by Government and the professional bodies with credit hours through TCMC to discuss the rational antibiotic usage for the elimination of AMR
10. Formation of Institution level Infection Control Committee for reviewing hospital infection control practices and rational antibiotic prescription patterns
11. The essential Drug List to be optimized as per the state antibiogram and separate antibiotic template for PHS, CHC & FRUs to be prepared and implemented
12. The State core committee to register as a Forum initially and a society later with the Chairman from the industry on a term rotation basis. The society shall chart specific road map for scientific scrutiny and research in the field of AMR
13. In future, State government to provide financial assistance through Plan budget to entertain rational antibiotic usage in public and private facilities & for surveillance & research activities about for microbiological resistance patterns
14. Upscale existing research under leadership of technical advisor
15. Annual national symposium each year to disseminate the findings of Good Antibiotic Prescription Practices
16. Recognition of success through an awards system for better performing professional bodies and institutions. Award criteria to be developed
17. Empirical evidence-based guidelines to be disseminated through a mobile application for smartphones
18. Curriculum on ASP to be developed for undergraduates and post graduates
19. Selected hospitals to develop institutional antibiograms which will serve as a base for the state antibiotic rational usage strategy
20. Establish clinical audits and surveillance

### State-wide education and training

In collaboration with the Kerala University of Health Sciences (KUHS), the PPP developed recommendations for revising the curriculum of undergraduate, house surgeons and post graduate trainees at government and private medical colleges. These recommendations were presented to the respective Board of Studies and Universities Academic Council. The aim of these revisions was to offer uniform training to provide academic and practical knowledge and training on rational antibiotic prescribing and Infection Prevention at all levels. The objective of recommendations was to enable clinicians to adopt strong and robust evidence based infection control behaviours and to independently care for patients. The new curriculum included four hours of additional training for undergraduates during their third year placement training, ten hours of training during the induction program for trainee surgeons, and 14 h of department specific training during the induction program of post-graduates. The outline of the course is provided in Box 2. Training material was developed to meet local epidemiology and clinical needs [44]. Multiple brain storming sessions for educational content development were conducted with domain experts from professional societies. Teaching modules integrated basics of microbiology, pharmacology, disease based clinical guidelines, antibiotic policy and IPC measures, designed with feedback from international experience to realize a one-way training programme for health care workers.

**Box 2:** Suggested curriculum change in medical schools

Module I:
1. Introduction to AMR
2. Infection Prevention and Control Basics
3. Good Antimicrobial Prescription Practices
Module II:
1. Application of PK/PD principles at bedside
2. Syndromic based Empiric therapy—Community Onset infections & Healthcare associated Infections
3. Interpretation and Use of Microbiological culture reports in clinical decisions
Module III:
1. AMS and its application at Individual Prescriber level
2. AMS and its application at organisational/departmental level
3. Evidence based IPC in specific clinical Units (ICU, OT, Dialysis etc.)
Module I &II for MBBS Undergraduates students
Module I, II&III for clinicians/Post Graduate students

All trainees had to pass an examination with a pass rate of 80%. Similar strategies have now been introduced to the curriculum in nursing, pharmacy and dental schools. A pilot is being conducted at one of the private medical colleges to provide learning for state-wide implementation in collaboration with the respective professional councils.

Additionally, 102 clinicians from different specialties representing hospitals across the state were invited to participate in a train-the-trainer initiative on a voluntary basis [45]. The aim was to train the clinicians so that they go back to their hospital and/or district and train their colleagues, covering all 14 districts of the State. The training was divided into three sessions covering the basics of microbiology, pharmacology of antibiotics and AMR, empirical therapy, including surgical prophylaxis, and infection prevention and control. Case scenarios, pertaining to the topic of the session, were also discussed with the participants to improve their practical skills. Formal certification for this training is provided from the Government and Kerala state Medical Association.

### Lessons learnt from the Kerala PPP for AMS

In this overview, we provided information on the strategy, advocacy and policy level efforts that were undertaken to establish a state and tertiary care level PPP for AMS. Multilevel strategies are essential to the successful implementation of AMS programmes. Working on developing an AMR containment strategy, exposed the magnitude of the AMR problem across the State. During the course of co-designing such strategies, a balance must be achieved in addressing the priorities across Human Health, Animal Health and the Environment. At Kerala, we worked on developing strategies across the One Health spectrum with equal emphasis. Attempts were made to collectively address the barriers of over the counter pharmacy shops, noncompliance of H1 prescription law, unwarranted antibiotic use in animal husbandry, making antibiotic prescription a legislative issue, poor continuous medical education, non-accredited microbiology laboratories and poor infrastructure, and sub-optimal infection control practices. The goal of having a strong and robust policy document along with an all-inclusive implementation plan was never lost. In order to do this, we had to cast the net wide, and this was only possible through the PPP approach.

Key strengths of the PPP were in the collaborative work of 18 professional medical societies to formulate Clinical Guidelines on Antibiotic Prescription; strong political will and leadership within the State; participation from human, animal and environmental departments; engagement of civil societies; and the constitution of a task force to monitor the ongoing work.

Turning the PPP initiative from a concept to implementation faced key challenges, which included: the effort to bring various groups (public and private) together; initial criticism of the initiative as there was no data to support the cause; changes in political leadership changing the priorities and delaying ownership of the programme; change in goal posts viz human health vis a vis One Health Approach; no dedicated funding available; bringing all stakeholders at one platform and agreeing on priorities / methodologies; lack of regulatory approach towards stewardship programme implementation; and lack of dedicated staff in public or private sectors to work on this initiative.

**Fig. 2:**
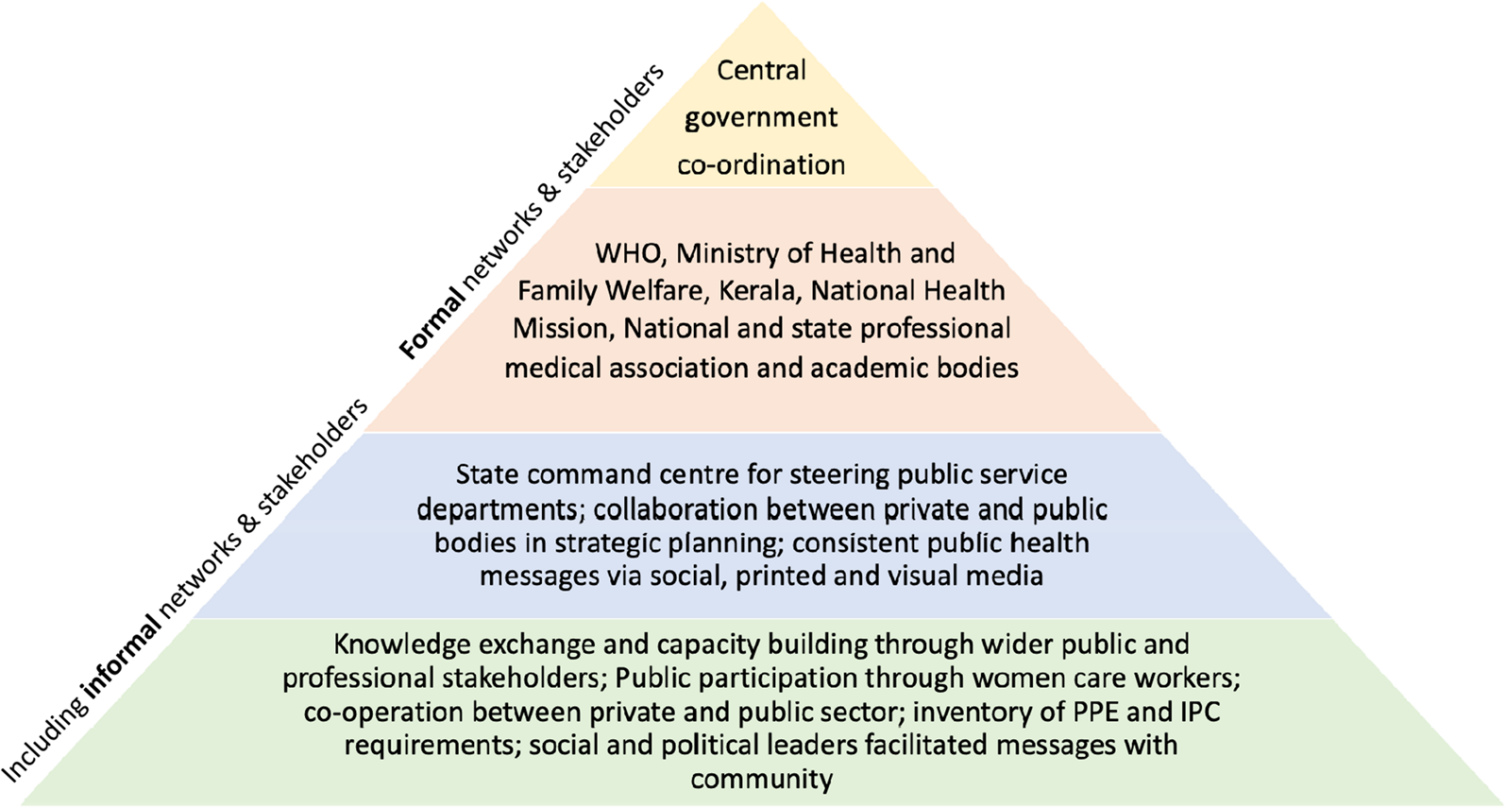
Lessons learnt from PPP and co-designed AMS initiatives at Kerala

Strong leadership pays dividends. In Kerala, the support from the government officials in the ministries of health was critical. There was a close coordination among all concerned public service departments and State Health Services, Medical Education, Drug Controller and Medical Services Corporation Ltd. The State Medical Association which maximized the private healthcare professionals’ participation. All professional societies were deeply invested into the strategic planning and drafting of the policies and developing the strategies. Collaborative work of multi-professional groups through regular meetings helped in co-design and development of disease based clinical treatment guidelines and state-wide infection prevention policy. Knowledge exchange though international and national platforms in the form of workshops for sharing of best practices is critical to success. Capacity building at both public and private tertiary care institutions included addressing practical and local solutions to the barriers e.g. good antibiotic prescription practices and infection prevention at all levels. (Fig. 2).

Despite limited financial support, the drive and will of each stakeholder motivated the teams and led to creation of history in being the first State in drafting clinical guidelines for antibiotic prescription, revising curriculum in medical schools, training of faculty and staff at public and private institutions and establishing a surveillance network.

### Limitations

This manuscript describes the strategic, policy, and advocacy that was required to initiate a PPP for AMS in the state of Kerala, which remains an atypical state for health standards in comparison to the rest of India. As such the experiences and processes described here may not be applicable to other states. The lack structural and process measures to tackle AMS meant that it was not possible to measure practices effectively. Now that the structures and mechanisms for the PPP for AMS have been established, the next phase is to evaluate this initiative. This is the first phase of the PPP initiative and was initiated in started in public and private tertiary care settings because the problem is greatest in tertiary care, and the resources available for such an initiative were more readily accessible. Furthermore, the problem is not yet quantified in secondary and primary care. If sustained success is demonstrated in tertiary care, the initiative will be expanded to other settings.

## Conclusion

The State of Kerala was the first in India to have developed a strategic approach to AMR including empirical clinical guidelines for infection management, implementation of an education programme for under- and post-graduate medical trainees as well as a robust State-wide training program covering 50,000 + general practitioners. In LMICs, where resources may be limited, the private and public sectors can work together successfully to develop strategies for addressing public health, including AMR. The Kerala PPI model AMR containment and IPC strategic plan has proved to be a successful model which has led to sustainable and multidisciplinary change. The model has delivered a State-level Antimicrobial Resistance Strategic Action Plan, laboratory based surveillance system and a healthcare associated infection based surveillance program to assess the performance of IPC and AMR containment activities. The lessons learnt from Kerala AMR strategy can provide learning for other States and countries aiming to implement action plans for AMR.

## Data Availability

Data sharing is not applicable to this article as no datasets were generated or analysed during the current study.

## Abbreviations

AMR: Antimicrobial resistance
LMICs: Low- and middle-income countries
PPP: Public private partnership
KARSAP: Kerala Antimicrobial Resistance Strategic Action Plan
NAP-AMR: National Action Plan on AMR
HAI: Healthcare acquired infection
AMS: Antimicrobial stewardship
DRI: Drug Resistance Index
MVT: Medical value travel
RNTCP: Revised National Tuberculosis Program
ASHA: Accredited Social Health Activist
GAPP: Good Antibiotic Prescription Practices
